# Determinants of Physical Health Outcomes in Individuals With Schizophrenia, Schizoaffective Disorder, and Bipolar Affective Disorder

**DOI:** 10.1192/bjo.2024.236

**Published:** 2024-08-01

**Authors:** Ella Rubinsztein, Kimberley Kendall

**Affiliations:** Cardiff University, Cardiff, United Kingdom

## Abstract

**Aims:**

Individuals with schizophrenia, schizoaffective disorder, and bipolar affective disorder have higher rates of cardiometabolic disease and have a reduced life expectancy compared with healthy controls. These mental health conditions are highly heritable and neurodevelopmental copy number variants (CNVs) are known to increase the risk of these disorders. Neurodevelopmental CNVs have also been associated with a range of cardiometabolic disorders. The aim of this research was to examine the relationship between neurodevelopmental CNVs and cardiometabolic disease in individuals with schizophrenia, schizoaffective disorder, and bipolar disorder.

**Methods:**

Using data from the UK Biobank, a group of individuals with schizophrenia, schizoaffective disorder and bipolar affective disorder was defined (n = 2,611) based on first-occurrence data. CNVs had previously been called using PennCNV and a set of 53 neurodevelopmental loci annotated. I carried out association analyses between neurodevelopmental CNVs and cardiometabolic disease phenotypes using logistic regression with age and sex as covariates.

**Results:**

There was a higher frequency of ischaemic heart disease, hypertension, obesity, and type 2 diabetes mellitus in individuals with schizophrenia, schizoaffective disorder and bipolar disorder than in controls. 2.1% of individuals with these mental health conditions carried a neurodevelopmental CNV. Carrying a neurodevelopmental CNV was significantly associated with type 2 diabetes mellitus (OR = 1.94, 95% CI 1.09–3.57, p = 0.025). However, this result did not survive Bonferroni correction for four tests (p value threshold 0.0125). I did not find any mediators of the neurodevelopmental CNV – type 2 diabetes mellitus association (of obesity, hypertension, cognition, smoking and socioeconomic status).

**Conclusion:**

The relationship between neurodevelopmental CNVs and type 2 diabetes mellitus should be examined in independent samples.

